# Binding of volatile aroma compounds to can linings with different polymeric characteristics

**DOI:** 10.1002/fsn3.526

**Published:** 2017-11-12

**Authors:** Xiaorong You, Sean F. O'Keefe

**Affiliations:** ^1^ Global Research The Coca‐Cola Company Atlanta Georgia; ^2^ Department of Food Science and Technology Virginia Tech Blacksburg Virginia

**Keywords:** can lining, flavor scalping, polymers, SPME

## Abstract

Flavor compounds have been shown to interact with packaging materials either by scalping, the movement of flavorings from the food product to the package, or by flavor release, movement of flavorings from the package to the food. Work has elucidated the parameters important for the scalping of flavor compounds to polyolefin packaging materials, but very little work has been conducted examining the scalping of flavor compounds by can lining materials. Can linings composed of three different polymers, polyolefin, acrylic, epoxy, were studied for binding of volatile flavor compounds (octanal, nonanal, decanal, eugenol, *d*‐limonene) at room temperature over a 2‐week period. Solid phase microextraction (SPME) was used with gas chromatography mass spectrometry to identify and quantify volatile compounds. Flavor compounds were studied at concentrations around 4–1,000 ppb. Fourier transform infrared spectroscopy was used to verify can lining polymer chemistry. Almost complete binding of all five of the volatile compounds studied was observed over 9–14 days at room temperature for each of the can lining chemistries. The number of time data points limited our ability to determine the order and rate constants of binding. This model system appears to be a valuable for investigating flavor binding of polymeric can lining materials.

## 1 INTRODUCTION

Packaged foods undergo changes in chemical composition during storage as a result of two‐way movement of small molecules between the package and the food. Aroma scalping refers to the movement of aromas from food to a package, and has been widely studied in citrus juices. Movement of small molecules from package to food may also occur, and this may result in off aromas or unwanted compounds being present in a food. Cork taint is a well‐known off aroma that results from aroma molecules (2,4,6‐trichloroanisole) moving from cork to wine, resulting in musty off flavored wine (Buser et al. 1982; Silvia Pereira et al. 2000). An example of binding or entrapment of aroma molecules by a food package is the scalping of limonene by plastic packaging (Fayouz, Seuvre, & Voilley, 1997a,1997b; Sadler & Braddock, 1990, 1991).

The binding or entrapment of aroma molecules by a package occurs initially by mass transfer of the aroma molecule to the package surface (adsorption), from the surface into the film itself (diffusion) and in cases with a fully permeable package, desorption to the outside of the package (Fayouz et al., 1997a; Figge, 1980). At initial stages, binding is a surface phenomenon. If the molecule absorbs into the material itself, a second phase of permeation occurs.

Aluminum cans are a convenient, light‐weight, highly recyclable, and robust package for beverages (Hosford & Duncan, 1994). They provide better light protection than glass and better oxygen and gas protection than plastics. The Coors company was an early adopter of seamless 2‐piece aluminum cans for beer and it was introduced by the Hawaii Brewing Company in 1958. Although Coca‐Cola had used cans as early as 1955, these were 3‐piece steel cans; aluminum cans were adopted by Coca‐Cola in 1967.

Metal cans require a lining to protect against food contact, especially with low‐pH foods in aluminum cans. Low‐pH solutions will corrode and dissolve aluminum, requiring a barrier layer. The main types of polymers used as can linings include epoxy, vinyl, acrylic, polyester and oleoresin (Bomgardner, 2013) and much research has been devoted to developing new linings that have required characteristics (Patel & Golden, 1987). Can linings must adhere well to walls, be stable during processing, resist corrosion, be flexible and not brittle, not degrade in the presence of acidic foods/beverages, and ideally be applicable to all food types (LaKind, 2013). The can lining with the best performance has been the epoxy lining (Bomgardner, 2013).

New can lining polymers must be tested for compatibility with foods or beverages stored in the lined cans as possible problems with color, corrosion, or aroma scalping may occur with novel linings. Beverages are often composed of hundreds of different aroma compounds, of which a dozen or two may be key contributors to the overall aroma of the product, with combinations of different compounds sometimes being required to evoke a particular flavor. Scalping does not occur across the board, so when scalping occurs in a food product, distortion of the aroma may occur, creating an unbalanced aroma profile (Fayouz et al., 1997a). This is particularly problematic as it is not always clear which compounds will scalp into which polymeric materials (Fayouz et al., 1997b). The objective of our work was to develop a model system to quantify scalping of aroma compounds into can linings and to compare scalping of model odorants in commercial can linings with different chemical characteristics.

## 2 MATERIALS AND METHODS

### 2.1 Aroma compounds and analysis

Eugenol, *d*‐limonene, octanal, nonanal, and decanal were obtained from Fisher Scientific (St. Louis, MO) and were stored under nitrogen gas at 4°C until use. Aroma compounds were dissolved in acidified (O‐phosphoric acid to pH 2.5) reverse osmosis water overnight at room temperature protected from light at stock concentrations (1.7–2.1 ppm). Stock solutions were diluted before use to working solutions of 4.1–4.2 ppb for all compounds but eugenol, which was diluted to ~1000 ppb. The higher concentration of eugenol was required for sensitivity of analysis.

A Shimadzu QP2020 Ultra GCMS was fitted with a SH‐Rxi‐5MS 5% dimethyl, 95% dimethyl polysiloxane column operated using helium carrier gas at 30 cm/sec. Injector temperature was 250°C and transfer line was 200°C. Oven was operated isothermally at 135°C. A split ratio of 1:10 was used because this provided better peak shape and reproducibility than splitless injection.

**Figure 1:**
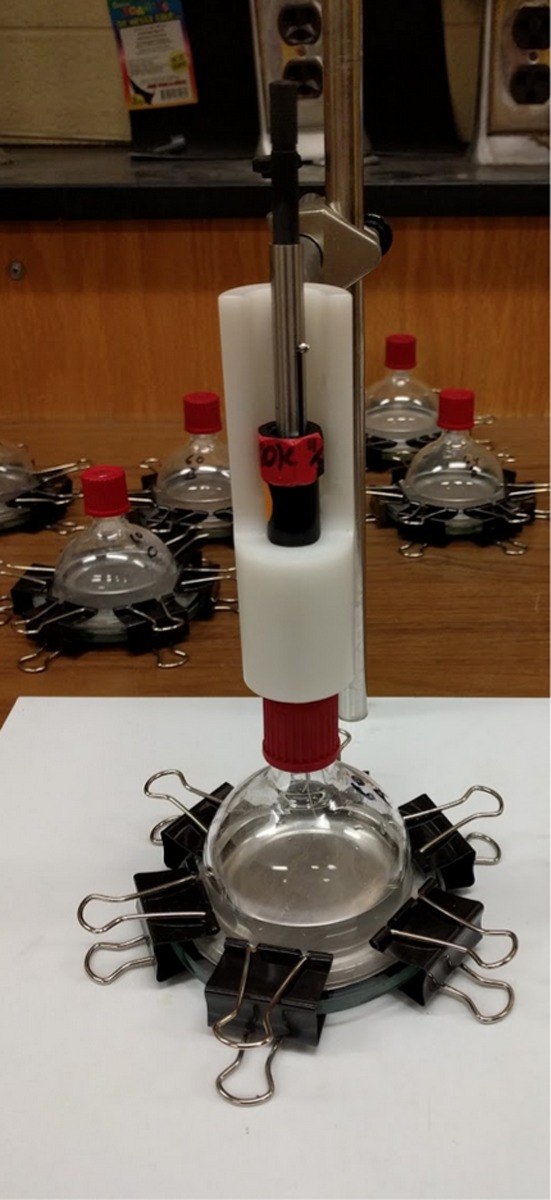
Reaction vessel used for aroma binding experiments

Two‐part glass reaction vessels were constructed to evaluate binding of aroma compounds to the can linings (Figure 1). Top parts were ~75 ml in volume, dome shaped, with a flange on the bottom and a removable screw cap with 13 mm septum on top. A round glass piece placed on the bottom allowed a circular piece of can lining to be fitted between the glass bottom and the flange of the domed top; these were clamped with large metal paper clamps. Reaction vessels were designed to allow similar volume to surface contact as a typical 12‐oz can.

Binding of aroma compounds to can lining was determined by measuring the headspace concentrations of the volatiles. Headspace concentration is related to the free concentration in the liquid phase. As binding occurs to the can lining, the free concentration in the aqueous phase is reduced and the headspace concentration is likewise reduced.

Solid phase microextraction was used to determine the concentration of volatiles in the headspace of the reaction vessels. StableFlex 2 cm fibers coated with divinylbenzene/Carboxen/polydimethylsiloxane (part number 57348U) were obtained from Sigma‐Aldrich Supelco. Fibers were conditioned in the GC injection port for 30 min (250°C) before use.

Teflon‐lined silicon septa were obtained from Agilent (Santa Clara, CA). They were fitted into the reaction vessel and the vessels assembled with round pieces of can with lining side up. After clamping together, the assemblies were loaded with 40.0 ml of aqueous aroma solution. Vessels did not leak unless carbonated beverages were used, where the pressure forced some liquid between the seams. Septa were first pierced with a standard beveled needle (Hamilton 10 μl) to create an easier entry for the SPME needle. SPME fibers were exposed to the headspace for 20 min at 22°C. Fibers were inserted into the injection port of the GCMS and left for 5 min. The gas chromatography column was operated at 135°C. Chromatograms were collected and total ion chromatograms used to determine aroma compound peak area. Mass spectra were used to verify peak identity using a Wiley library. Two or 3 replicate reaction vessels were used with each aroma compound, can lining combination. After the fiber was removed, the septum was quickly replaced on the reaction vessel for repeated measures analysis at other times. Headspace concentrations were determined each 3–4 days until less than 10% of the original area was present.

### 2.2 Cans for analysis

Four separate sets of empty cans were used for testing. They were commercial samples that had epoxy (a), polyolefin (b) or acrylate (c, d) linings. Linings are proprietary and closely guarded; additional specifics on the exact nature of the chemistry is not available.

### 2.3 Reflectance FTIR

Reflectance FTIR spectra were obtained from can linings to validate polymer characteristics. A Fourier Transform InfraRed (FTIR) spectrometer (Thermo Scientific Nicolet 8700, Madison, WI) equipped with an attenuated total reflectance (ATR) crystal was used to collect absorbance of the can linings. A blank background spectrum was collected before every sample. The range of spectra was from 4000 to 500 cm^−1^ with 96 scans and a resolution of 4 cm^−1^.

## 3 RESULTS AND DISCUSSION

Most of the work examining aroma scalping by plastic food packages has focused on plastic bottles (Fayouz et al., 1997a,1997b; Johansson & Leufvén, 1997; Sadler & Braddock, 1990, 1991; Sajilata, Savitha, Singhal, & Kanetkar, 2007). Recently, Wietstock, Glattfelder, Garbe, and Methner (2016) examined aroma scalping by crown cork liners and can linings from different manufacturers.

The concentration range used for this work was selected to be near the limit of detection for the GCMS. A given amount of can lining will have a theoretical maximum adsorption and diffusion that can occur. Concentration used will impact the relative amount of the volatile that will be bound by the polymer. Thus, if a high concentration is used, sensitivity for relative affinity for the polymer will be low. On the other hand, the maximum amount that can be bound (absorption plus diffusion) will not be measurable. Our model systems were designed to look at relative rates of sorption of single aroma molecules at very low concentrations. In any case, a real world flavoring would be composed of hundreds of aroma compounds that may compete with one another for binding. The concentrations for *d*‐limonene, octanal, nonanal, and decanal were 4.1–4.2 ppb. The concentration for eugenol was 1,006 ppb. The large difference in concentrations was necessary due to sensitivity difference in the SPME‐GCMS headspace analysis.

Reaction vessels were manufactured by the Virginia Tech glass laboratory for these experiments. The average volume of the vessels was 76.6 ml ± 0.21 (mean, standard deviation). The diameter at the flange edge is 86 mm, the height 65 mm and the exposed surface diameter 61 mm. With 40 ml aroma solution used per vessel, the volume to surface ratio is 2.1 ml/square cm.

**Figure 2:**
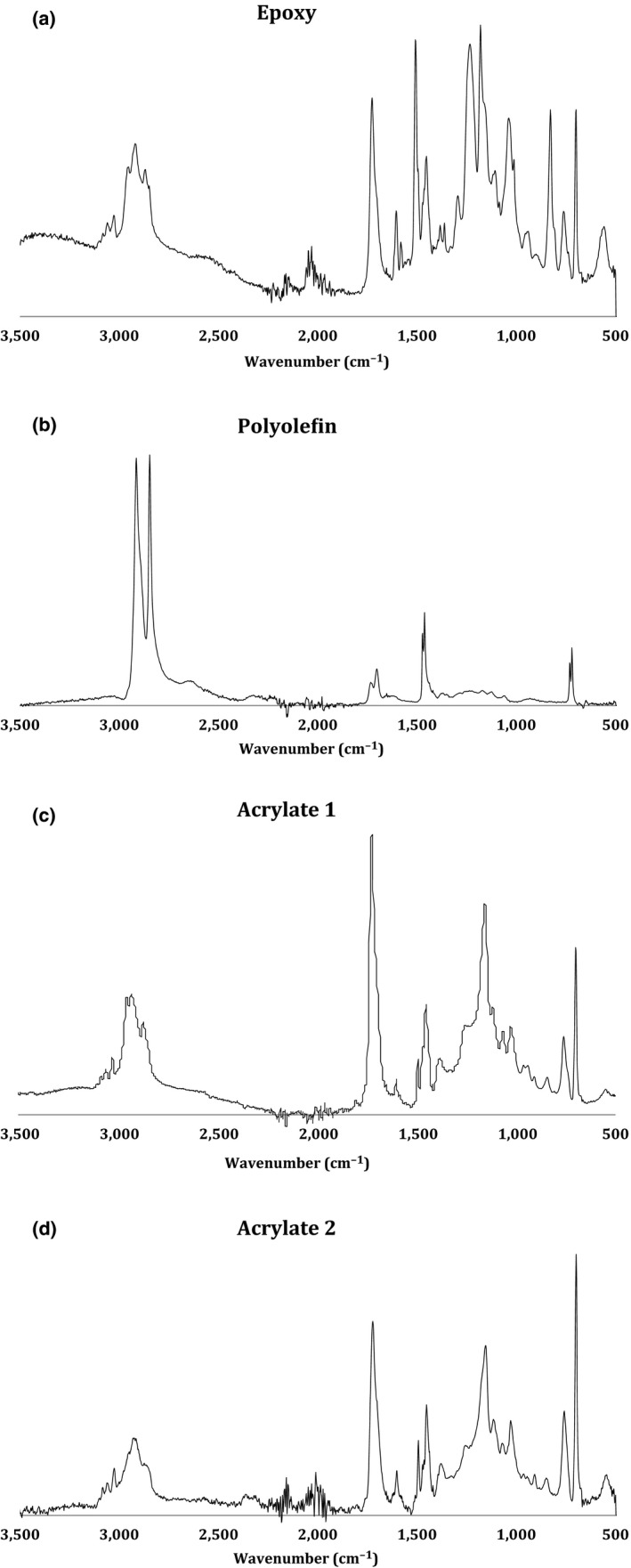
FTIR reflectance spectra of can linings used in this work

The FTIR spectra of the can linings are shown in Figure 2a–d. The FTIR spectra showed characteristic bands for chemical bonding but we did not attempt to assign structural information to the absorbance bands, rather we used this information to ensure our polymer assignments were correct (Manfredi et al., 2005). For example, polymer A closely resembles DGEBA (diglycidylether of bisphenol A) (González González, Cabanelas, & Baselga, 2012). Polymer B resembles polyethylene (http://www.ftir-polymers.com/soon_soubory/image011.gif) and C and D resemble polymethmethacrylate (http://www.ftir-polymers.com/soon_soubory/image033.gif). The chemical assignments via FTIR agree with the designations from commercial suppliers, so we are confident of the nature of the polymers.

Initial experiments were conducted to determine the variability in replicate fibers and two fibers were selected for analysis that had the closest binding affinity for limonene. Variability within fibers was less than 1%.

**Figure 3:**
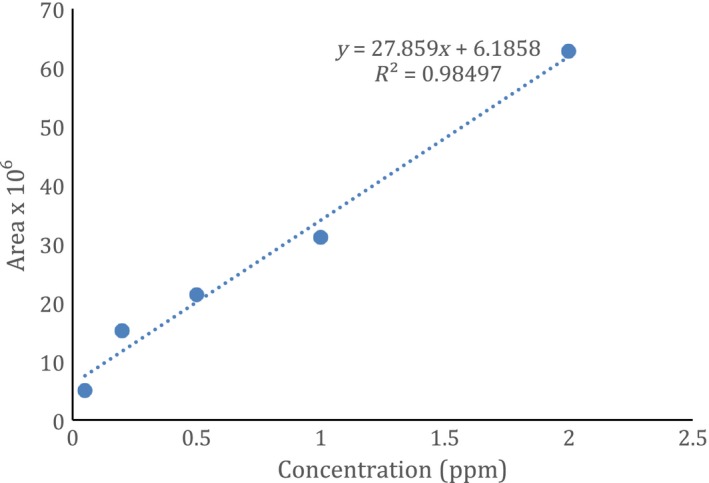
Standard curve for *d‐*limonene using described model system

Can linings were carefully cut to fit the reaction chambers to ensure than no fingerprints, marks or damage (crinkling) to the lining took place. The lining was exposed to the acidified water containing aroma compounds for a minimum of 2 hr before day 0 analysis. A typical concentration‐area curve for limonene is shown in Figure 3.

Data for time zero (obtained within 3 hr of equilibrium time, within 4 hr of reaction vessel loading) indicated that there was no difference observed between can linings or a glass control (only glass on the bottom, no can lining). A typical average across treatments was 21.5 ± 0.38 (coefficient of variation 0.79%). There was a large difference in response between *d*‐limonene, the carbonyls and eugenol. Eugenol peak areas were about 350–400 times lower than the other compounds in the 2 ppm concentration range. To obtain similar signal to noise rations, a higher concentration of eugenol was used compared to the other aroma compounds.

**Figure 4:**
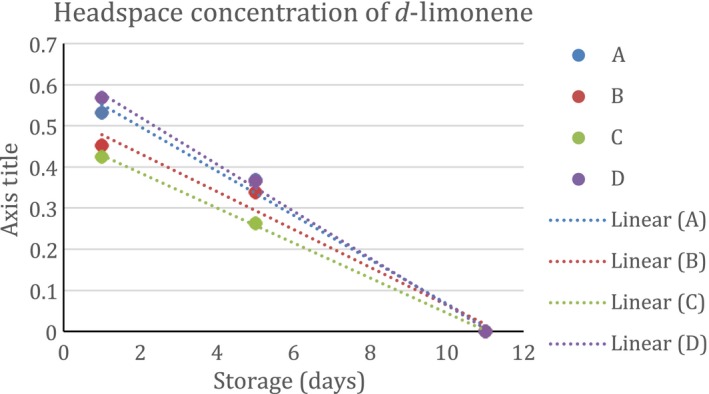
Headspace concentration of *d‐*limonene in model systems equilibrated with different can liners at room temperature

The headspace concentration of *d*‐limonene in the presence of the four different can linings is shown in Figure 4. There was almost complete scalping of the limonene by 11 days of storage. The scalping has two phases, first adsorption to the surface, and second diffusion into the polymer. We only had three data points for this scalping; scalping was complete by 11 days (we had expected it to take longer). The amount of limonene scalped equals 8.8 ng per square cm of surface.

Wietstock et al. (2016) reported limonene equilibrium binding to low‐density polyethylene occurred within 15–20 days at room temperature. Similar times to reach equilibrium were reported for myrcene, linalool and α‐terpineol, but time to equilibrium for caryophyllene, α‐humulene and geranol were longer. The volume to surface ratios were not reported in their study. Sadler and Braddock (1991) had earlier shown that limonene, myrcene and ethyl butyrate had much better affinity to low‐density polyethylene than did octanal, citral or linalool. They stated that solubility on the polymer was proportional to the diffusion coefficients observed. The concentration ratios between volatile and polymer are relevant to the rate and ultimate amount of scalping; model systems with a lower mass of volatile relative to the mass of the polymer would be expected to differ in scalping compared to model systems where the aroma compound has a higher relative mass ratio.

**Figure 5:**
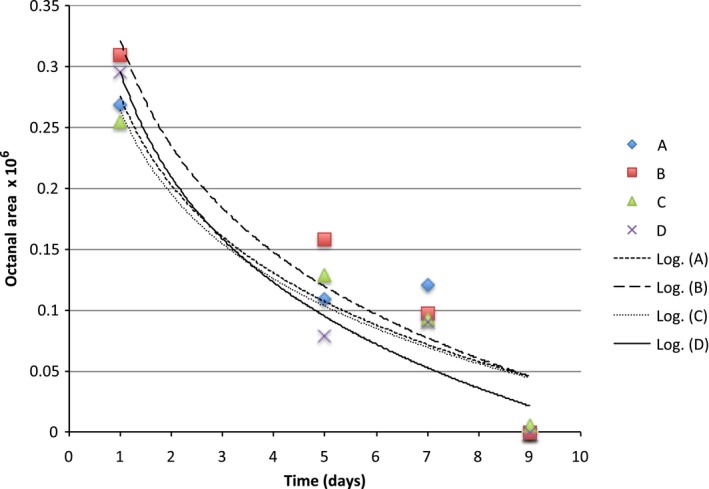
Headspace concentration of octanal in model systems equilibrated with different can liners at room temperature

**Figure 6:**
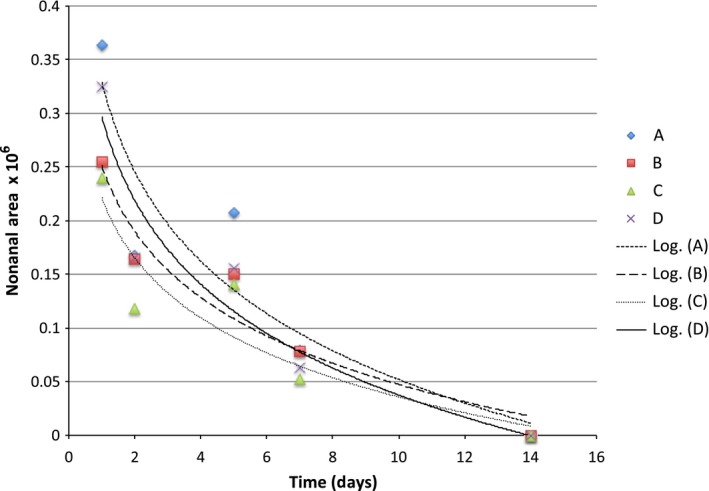
Headspace concentration of nonanal in model systems equilibrated with different can liners at room temperature

**Figure 7:**
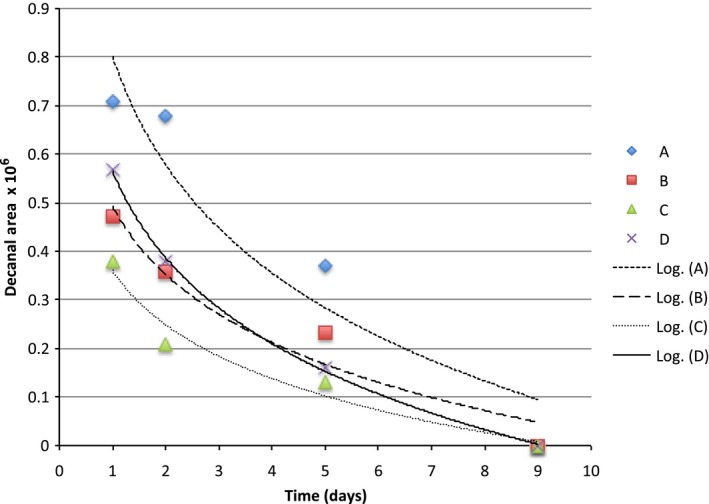
Headspace concentration of decanal in model systems equilibrated with different can liners at room temperature

**Figure 8:**
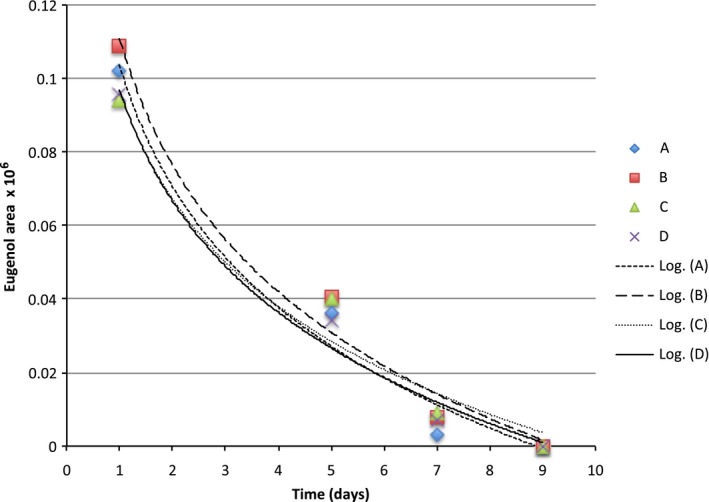
Headspace concentration of eugenol in model systems equilibrated with different can liners at room temperature

The binding curves for limonene were quite similar across can linings and we probably needed more sampling points to observe an expected logrithmic diffusion (Figures 4, 5, 6, 7, 8). The slopes were quite similar to one another (*p* > .05) and the headspace concentration of *d*‐limonene was not measurable by 11 days storage at room temperature. The characteristics of the lining polymers would be expected to have an impact on scalping rates and amounts. Till et al. (1987) pointed out that time, temperature (related to whether or not polymer was above or below the Tg, glass transition temperature), migrant concentration, agitation, polymer morphology (thickness) and migrant type affected scalping. Fayouz et al. (1997b) reported the free volume, crystallinity, polarity, tacticity, crosslinking, orientation (stretching), additives as well as the polymer blends affect rate of sorbtion of a given compound. Polymers that were low in crystallinity and/or above their *T*_g_ had greater scalping than those below the *T*_g_ or with higher crystallinity. We did not measure the *T*_g_, density, average molecular weight or other parameters that may be important for the can lining polymers.

Octanal, nonanal, and decanal were studied because they are a homologous series of carbonyls with importance in citrus aroma. Prior work has shown that addition of each methylene unit may increase the affinity of the molecules for nonpolar polymers.

The scalping of the aroma compounds all followed logarithmic behavior except for limonene. The reason for this could be limited data points due to the rapid scalping of the limonene from the aqueous matrix. A logrithmic decay (scalping) is expected based on previous studies and theory of diffusion (Fayouz et al., 1997b). By 5 days, about 50% of the original aroma molecules in solution were lost by absorption or diffusion into the plastic liner. Wietstock et al. (2016) studied movement of aroma compounds from beer into crown cap liners and can linings. They found that 100% of *trans*‐caryophyllene, 71.2% of myrcene, and 53.7% of limonene were found in the can lining compared to the aqueous beer at the single time period they examined (46 days). For linalool, only 0.4% of the compound was found in the liner, 99.6% remained in the aqueous solution. Alpha‐terpineol was not detected in the can lining and remained completely soluble in the aqueous component. This shows the clear importance of polarity in the adsorption and diffusion of volatiles into can lining. Unfortunately, the type of can lining polymer used in their study was not reported. For cold pressed orange oil in contact with LDPE, around 70%–80% sorption was reported at room temperature (Fayouz et al., 1997b). Cold pressed orange oil is mainly composed of *d*‐limonene, with expected concentrations over 90% of the total mass. In a model system such as LDPE exposed to orange oil, one expects a saturation to occur and also there may be cooperative effects where some limonene could affect the polymer in such a way as to increase the binding of additional limonene molecules. This is quite different from a model system such as ours where very low concentrations of the volatile are present.

Our results show that volatile aroma compounds do absorb/diffuse into different can lining polymers. The precision of the SPME‐GCMS procedure may not be good enough to make clear conclusions regarding which can lining polymer have greater scalping of the various aroma compounds. There appeared to be a more clear effect of can lining polymer on the binding of decanal than for other compounds. The scalping curves were more clearly separated from one another. The can lining polymers differed in chemical composition, as well as other important parameters we did not measure (thickness, crystallinity, etc.) that would probably impact scalping. All of the polymers examined resulted in almost complete scalping within a 7–14 day time period. One of the important aspects of our model system is that we used a very low concentrations of aroma compound for testing. We used this to avoid saturation of binding that may occur if the mass ratio of volatile:polymer is high. The amount scalped would depend on the affinity of the aroma volatile with the polymer, and this has been reported as simple solubility, which would be impacted by the mass ratios.

The change in distribution ratio from aqueous component to packing polymer can increase with each CH_2_ added to chemical structure, but in some cases it can also decrease (Sajilata et al., 2007). Generally, the greater the carbon chain length, the more nonpolar and the higher the affinity to nonpolar polymers such as polypropylene and polyethylene. Although linalool's higher solubility than limonene in polyethylene should facilitate its migration into the polymer matrix, Wietstock et al. (2016) found almost no migration of linalool into can linings compared to limonene. They reported the same thing in polyethylene crown cap liners, where linalool was not scalped (0.6% in liner, 99.4% in beer) and yet limonene was (87.9% in liner, 12.1% in beer).

Differences in experimental conditions will likely impact results observed. In our experiments, concentrations of volatile compounds were selected to push the sensitivity of the GCMS; 4.1–4.2 ppb for limonene and the aldehydes and 1,000 ppb for eugenol. Each was tested in acidified water alone, so there was no effect of other components such as caffeine, other volatiles, sweetener, colorings, etc. Whether or not these compounds could impact scalping is unknown. Competition of different aroma compounds would also be expected to impact scalping, but the specifics of this are unknown. Limonene is thought to co‐sorb and facilitate the movement of other aroma molecules into nonpolar packaging materials and sorption of volatiles into linear low‐density polyethylene is reported to be maximal at 25°C, and lower at 5°C and 75°C (Sajilata et al., 2007).

## 4 CONCLUSIONS

Eugenol, *d*‐limonene, octanal, nonanal, and decanal in the ppb concentration range were scalped from acidified aqueous model systems by epoxy, polyolefin, and acrylate can lining polymers. Further work is needed to clarify differences in scalping by can liners with different polymeric characteristics.

## CONFLICT OF INTEREST

None declared.
